# Nurse-Led Self-Management Support After Organ Transplantation – A Multicenter, Stepped-Wedge Randomized Controlled Trial

**DOI:** 10.3389/ti.2024.13175

**Published:** 2025-01-06

**Authors:** Regina van Zanten, Monique van Dijk, Joost van Rosmalen, Denise K. Beck, AnneLoes van Staa, Ann Van Hecke, Emma K. Massey

**Affiliations:** ^1^ Department of Internal Medicine, Erasmus MC Transplant Institute, University Medical Center Rotterdam, Rotterdam, Netherlands; ^2^ Department of Internal Medicine, Section Nursing Science, Erasmus Medical Center, Rotterdam, Netherlands; ^3^ Department of Biostatistics, Erasmus Medical Center, Rotterdam, Netherlands; ^4^ Department of Epidemiology, Erasmus Medical Center, Rotterdam, Netherlands; ^5^ Research Centre Innovations in Care, University of Applied Sciences Rotterdam, Rotterdam, Netherlands; ^6^ Department of Public Health and Primary Care, University Centre of Nursing and Midwifery, Ghent University, Ghent, Belgium; ^7^ Department of Nursing Director, Ghent University Hospital, Ghent, Belgium

**Keywords:** nurse practitioners, patient participation, motivation, goal, self-efficacy

## Abstract

In this unblinded multi-center stepped-wedge randomized controlled trial the effectiveness of the nurse-led ZENN-intervention was tested in promoting self-management skills in comparison to standard care among heart, lung and kidney transplant recipients. This intervention is based on behaviour change theories and was conducted in four sessions over 6 months at the outpatient clinic. The experimental group received standard care, plus the ZENN-intervention, while the control group received only standard care. Both groups completed questionnaires at baseline, at 6 months and 1 year follow-up. At baseline, the experimental group (n = 69) scored significantly lower than the control group (n = 106) on the primary outcome Skills and Technique Acquisition (STA). No significant between-group differences were found on the secondary outcomes self-management, self-regulation, quality of life and medication adherence at T1 and T2. There was a significant increase on the self-management scale STA between T0 and T1 in the experimental group. Therefore, participants included in the experimental group had lower self-management skills at baseline and reported significant improvement after completing the intervention. No significant intervention effect was found in the primary analysis, however, for recipients with reduced self-management skills the intervention may be beneficial.

**Clinical Trial Registration:**

https://onderzoekmetmensen.nl/en/trial/24150, Netherlands Trial Register NL8469.

## Graphical Abstract

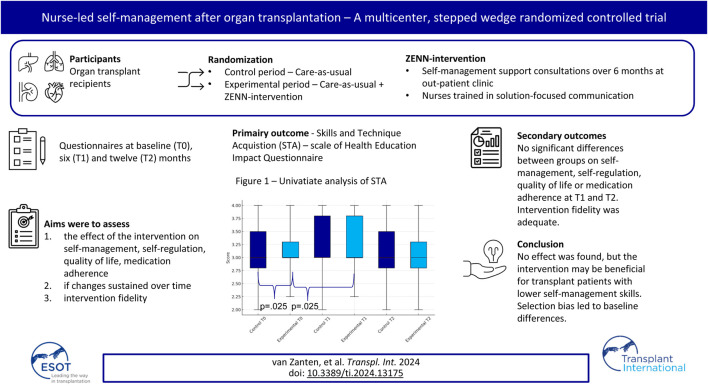

## Introduction

Life after a solid-organ transplantation (SOTx) can present medical, social and emotional challenges [1–8]. Recipients need optimal self-management skills to deal with these challenges. Self-management can be defined as “the individual’s ability to manage the symptoms, treatment, physical and psychosocial consequences and life style changes inherent with a chronic condition” [9]. Previous research has shown recipients’ need for holistic care after SOTx [10, 11]. According to recipients, support for medical management is sufficient, but emotional and role management support is often lacking [11, 12]. Optimal self-management can contribute to better clinical outcomes, lower healthcare costs [13] and a higher QoL [14].

Skills needed to achieve adequate self-management include awareness of possible problems, ability to solve problems, setting goals, making an action plan, executing it and being able to monitor and evaluate progress and, if necessary, adjust the goal. Many of these are self-regulation skills as defined by Self-regulation Theory [15]. Self-regulation can be defined as a “goal-guidance process, occurring in iterative phases, that requires the self-reflective implementation of various change and maintenance mechanisms that are aimed at task- and time-specific outcomes” [15]. Three phases are important here [1] goal selection, setting and representation [2]; active goal pursuit; and [3] goal attainment and maintenance or, when necessary, goal disengagement [15]. Adequate goal pursuit requires intrinsic motivation, self-efficacy, perseverance, planning and flexibility [16].

Previous research highlighted that there is a need for improved SMS in the first-year post-transplantation, but that attitudes, needs and preferences of transplant recipients regarding self-management vary per person [10, 17]. Current interventions have been criticized for not being able to provide person-centered and tailored support due to a one-size-fits-all approach. Moreover, interventions have been investigated specific patient groups with few studies addressing common self-management challenges among recipients of the various organs [18–20]. Furthermore, interventions are insufficiently guided by behavior change theories [20–22] and are time and resource intensive. To address some of the shortcomings, a SMS intervention was developed [17]. The overall aim of the ZENN-intervention (ZElfmanagement Na Niertransplantatie; Dutch acronym for self-management after kidney transplantation) is for recipients, with the guidance of nurse practitioners (NPs), to enhance their self-management skills in order to integrate their treatment and life goals. Key elements of the intervention are [1] a holistic approach [2], tailoring to patients needs and priorities [3], shared-decision making, and [4] patient empowerment. Early pilot-testing among kidney transplant recipients demonstrated feasibility and acceptability [23]. Given that self-management challenges and skills required after transplantation are comparable for recipients of kidney, liver, heart and lungs, the ZENN intervention may be beneficial for all SOTx recipients [20]. In this study, the first aim was to assess the effect of the intervention on participants’ self-management and self-regulation skills, QoL, medication adherence, controlling for socio-demographic and medical characteristics. The second aim was to assess if the changes were sustained over time and the third aim was to assess adherence to the intervention protocol by NPs to test the intervention fidelity.

## Materials and Methods

### Design

This multi-center study had an un-blinded stepped-wedge cluster randomized controlled trial (RCT) design and was performed between September 2020 and May 2022 [24]. A classical RCT with blinded group allocation was not suitable because it is not possible to expect NPs to switch between using and not using the learned communication techniques depending on group allocation. Additionally randomization was performed at the department level and not on NP level, due to the small number of NPs per department. All departments started with a control period and the start date of transition to the experimental period was randomized. The patients in both groups are different, which means that they will not cross-over from control to experimental group. Seven departments from five university medical centers in the Netherlands were included: four kidney transplant departments, one heart transplant department, one liver transplant department and one lung transplant department.

### Eligibility Criteria

Potential participants were eligible if they had received a heart, kidney, liver or lung transplantation, were over 18 years old, were transplanted two to 13 months ago, had sufficient understanding of the Dutch language and had a functioning graft. Exclusion criteria were: cognitive limitations, participating in other lifestyle or self-management promoting programs which could influence the outcome and in case of kidney transplant recipients, renal replacement therapy expected to be needed within 3 months of inclusion.

### Procedure

The intervention was delivered at the out-patient clinic by NPs. Immediately prior to transition from the control period to the experimental period, NPs were trained in the theoretical background and practical steps in carrying out the intervention. This training consisted of an e-learning course and a live training guided by a psychologist using a training actor to practice communication skills. The live training was conducted online due to COVID-19 restriction at the time. Participants completed a baseline (T0), a 6 months follow-up (T1) and a 12 months follow-up questionnaire (T2). Participants in the experimental group received the intervention between T0 and T1. The CONSORT Guidelines were used to guide reporting [25].

### ZENN-Intervention

The ZENN-intervention [17] is a nurse-led SMS intervention primarily based on the theoretical framework of the Self-Regulation Theory. The intervention strategies are based on evidence-based techniques taken from Self-Regulation Theory [15], Solution-Focused Brief-Therapy [26, 27] and Motivational Interviewing [28].

**FIGURE 1:**
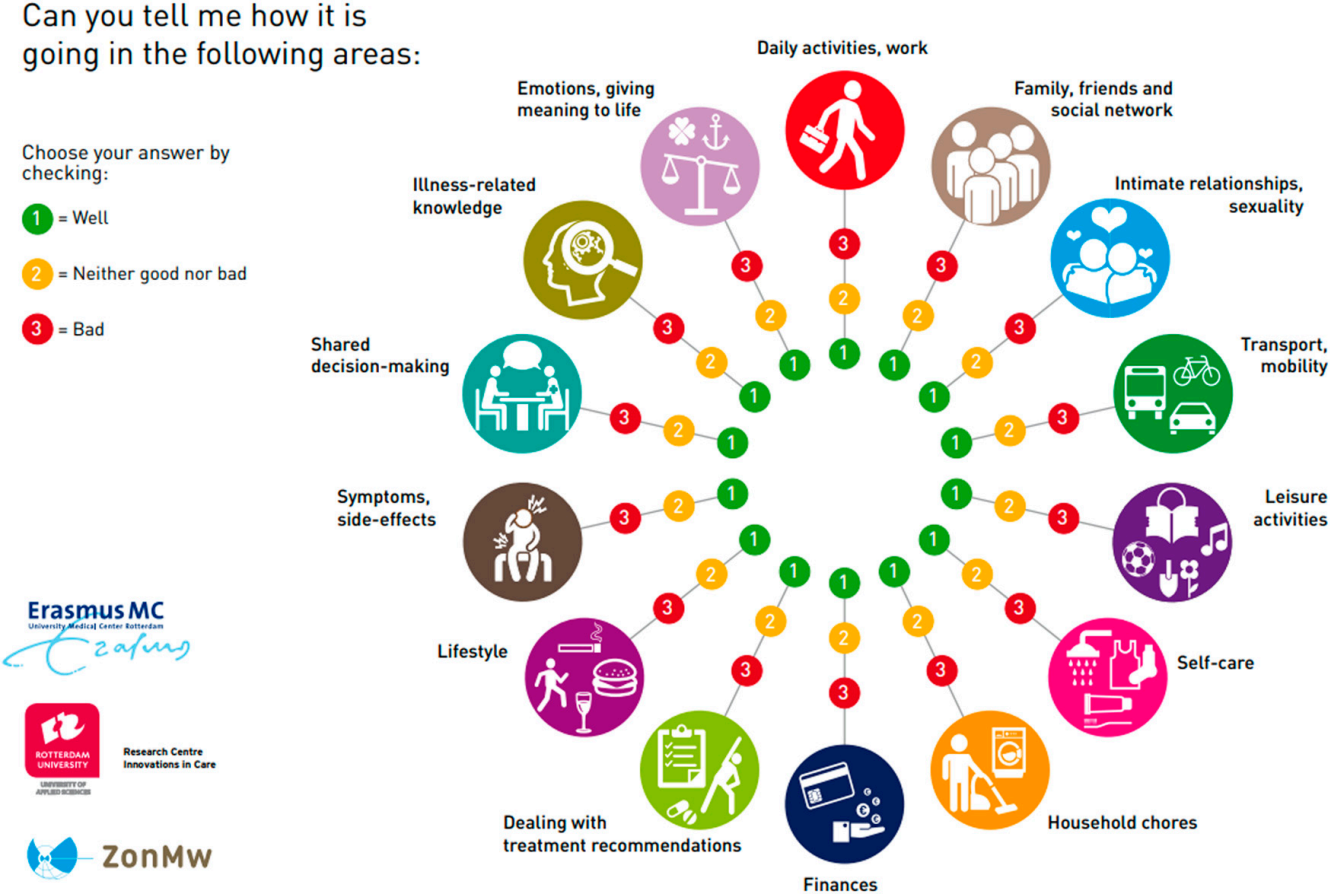
Self-Management Web.

**FIGURE 2:**
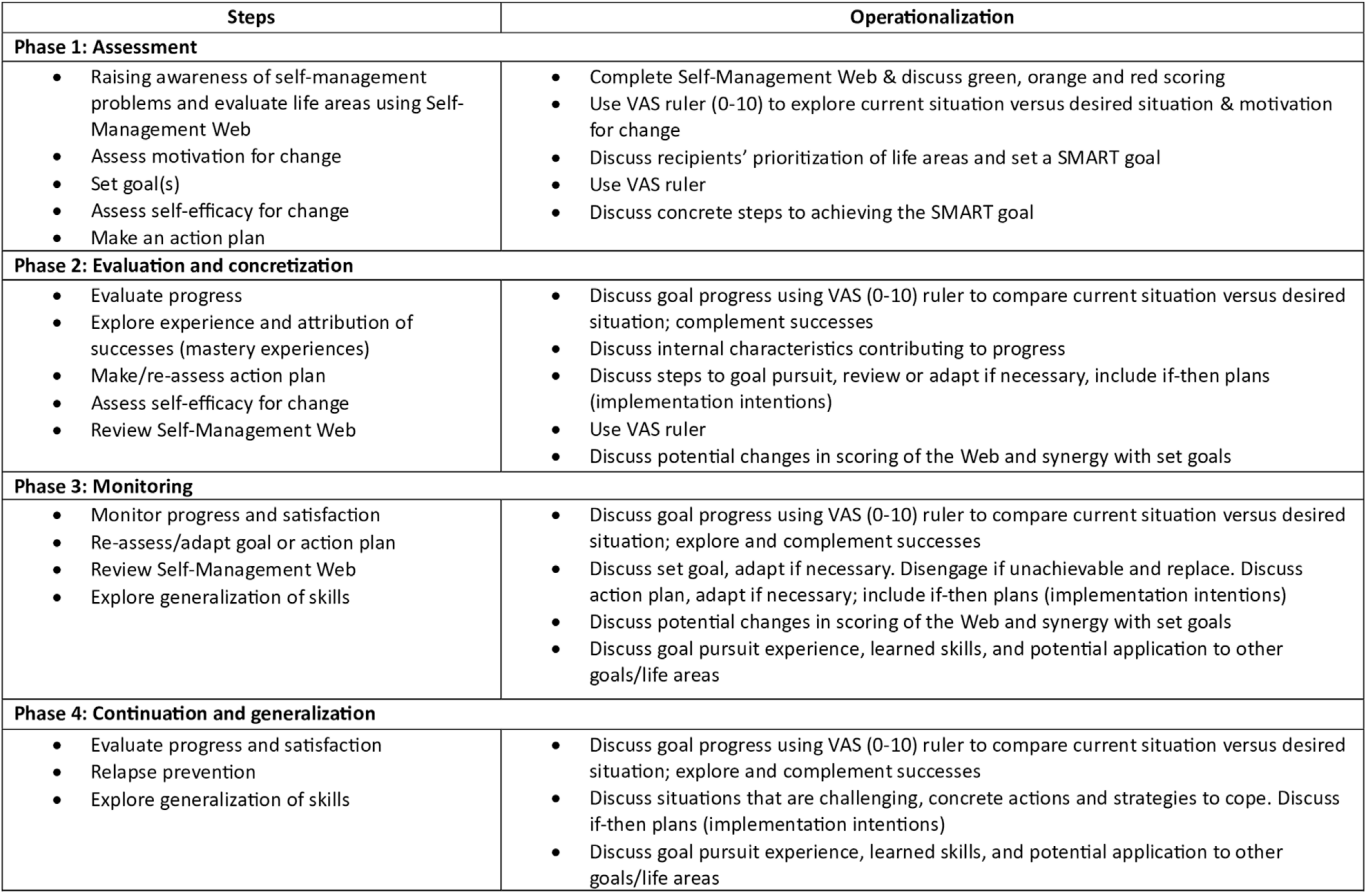
Content of phases ZENN-intervention. Adapted from Beck et al. [17]. Abbreviations: VAS, Visual Analogue Scale; SMART goal, Specific, Measurable, Achievable, Relevant and Time-Bound.

The intervention is divided over several approximately 15-minute consultations. The intervention has four phases that must be completed, whereby the number of consultations depended on the logistical constraints of the setting and needs of the patient. Tools used during the consultations are the communication aid Self-Management Web (Figure 1) and a logbook in which the NP can keep track of the stages completed. For a visual overview of the steps and operationalization per phase, see Figure 2. The development of the ZENN-intervention and pilot testing has been extensively described elsewhere [17, 23].

### Data Collection

#### Primary Outcome

*Self-management* was measured using the 40-item Dutch Version of the Health Education and Impact Questionnaire (heiQ) [29]. This instrument consists of eight domains. As there is no overall score of the heiQ, the “Skills and Technique Acquisition” (STA) subscale was chosen as primary outcome. This scale was chosen as the content was deemed nearest to the skills promoted in the intervention. The other seven subscales are described below as secondary outcomes. Response options are based on a 4-point Likert scale: “Strongly disagree” [1] to “Strongly agree” [4]. Interpretation of the heiQ is through mean scores on each domain, with subscale scores ranging between 1 and 4. Good validity and reliability have been established [29].

#### Secondary Outcome

The remaining subscales of the heiQ are “Health directed activity,” “Positive and active engagement in life,” “Emotional distress,” “Self-monitoring and insight,” “Constructive attitudes and approaches,” “Social integration and support,” and “Health service navigation” [29]. Higher values on the domain indicate higher levels of self-management, with the exception of the scale “Emotional distress,” for which the interpretation is reversed.

*Self-regulation* was measured using the 21-item Self-regulation skills instrument in transplantation (SSIt) [30]. This instrument is divided into two scales “Setbacks” and “Successes.” Response options are based on a 5-point Likert scale: (1) “Completely disagree” to (5) “Completely agree.” Mean scores are calculated per subscale. A higher score on the subscale “Setbacks” indicates greater difficulties with the process of goal setting, initiating a plan to reach a goal, and dealing with setbacks. A higher score on the subscale “Successes” indicates successes in the process of goal setting, intrinsic motivation for initiating the plan, and self-efficacy. Good validity and reliability have been established [30].

*Quality of life* was assessed using the 26-items World Health Organization Quality of Life – Brief Version (WHOQoL-BREF) [31]. This instrument consists of five domains: “Physical health”; “Psychological”; “Social relationship”; Environment, and “Overall QoL” and “General health.” Mean scores are calculated per domain as well as for the overall QoL. A higher score on the scale(s) indicates a higher level of QoL. Good validity and reliability have been established [31].

*Medication adherence* was measured using the Basel Assessment of Adherence to Immunosuppressive Medication Scale (BAASIS) [32]. The BAASIS is divided into two parts. The first part consists of four questions with the answer options (0) “No” and (1) “Yes.” If “Yes” to any of these items, the patient is categorized as non-adherent. The second part than needs to be answered per item to indicate; how often they are non-adherent: (1) “Never” to (6) “Every day.” Good validity and reliability have been established [32].

The *evaluation of experience* with the intervention was measured at T1 using the 5-item subscale “Patient-centeredness” (Cronbach’s α = 0.83) of the American Consumer Assessment of Health Plan Survey (CAHPS) [33]. In addition, a visual analogue scale (1–10) was used to evaluate the overall experience of the nurse-led care. A higher score indicates a better overall experience. In addition, the participant was asked if they would recommend the ZENN-intervention to peers. Answer options were (1) “Yes, because…” and (2) “No, because…”

*Socio-demographic and medical characteristics* measured were gender, age, educational level, organ type and donor type. The donor type question was answered by NPs as participants are not always aware of the source of the organ.

*Intervention fidelity* was operationalized as adherence to the intervention protocol. Therefore, the NP completed a questionnaire about the number of consultations each participant received; how often the Self-Management Web was used; if each step of the intervention was completed and if the participant received the patient booklet. The greater the variation, the more likely intervention fidelity can be questioned [34]. A percentage of 80% per item was considered satisfactory.

### Sample Size and Power

In order to obtain a power of 80% to detect a significant effect of the intervention, 82 patients per group were needed [24]. To account for the effects of correction for covariates, dropout and missing data, and contamination, we aimed for inclusion of 100 patients per group.

### Ethical Considerations

The Medical Research Ethical Committee Erasmus MC approved this study protocol on 8th November 2019 (MEC number: MEC-2019-0671). The trial was conducted in accordance with the principles that have their origin in the Declaration of Helsinki 2013 and the principles of Good Clinical Practice.

### Data Analysis

The control and experimental group at T0 were compared on patient characteristics as well as primary and secondary outcomes. The outcome was compared within each group between T0 and T1, and between T0 and T2. Descriptive statistics were presented as frequencies for categorical variables. Continuous variables were described as mean and standard deviation for normally distributed data and median and interquartile range for non-normally distributed data. The primary analysis was a univariate analysis of the effect of the intervention. Continuous outcomes at T0, T1 and T2 were compared between groups and tested using the independent samples t-test for normally distributed data or the Mann-Whitney U test for non-normally distributed data. Within-groups comparisons of continuous outcomes were performed using Wilcoxon signed-rand tests. For the BAASIS, a 2 × 2 chi-squared test was conducted and a within-groups analysis was conducted using a generalized estimating equations (GEE) model. For the multivariable analyses, a general linear model for repeated measurements (GLM) was applied to account for group (experimental or control), time-point (T0, T1 or T2), the interaction between group and time-point, the covariates “type of organ” and transplant center and other significant covariates. The within-patient correlations between repeated measurements were modeled using an unstructured covariance matrix. In addition, the results of the general linear models were summarized using the estimated marginal means, which are the predicted values of the response adjusted for covariates. These estimated marginal means were compared between participants in both groups at T1 and T2. In case of a skewed distribution of the outcome, leading to non-normally distributed residuals in the linear model, the outcome was dichotomized. This dichotomized outcome was then analyzed using a GEE model with a logit link function and a binomial distribution (i.e., logistic regression for repeated measurements). Based on the intention-to-treat principle, all models were estimated using all eligible participants from whom data was obtained. Data imputation was used when missing data occurred, as recommended in the instrument manuals. A *p*-value <0.05 was considered statistically significant.

## Results

### Inclusion

**FIGURE 3:**
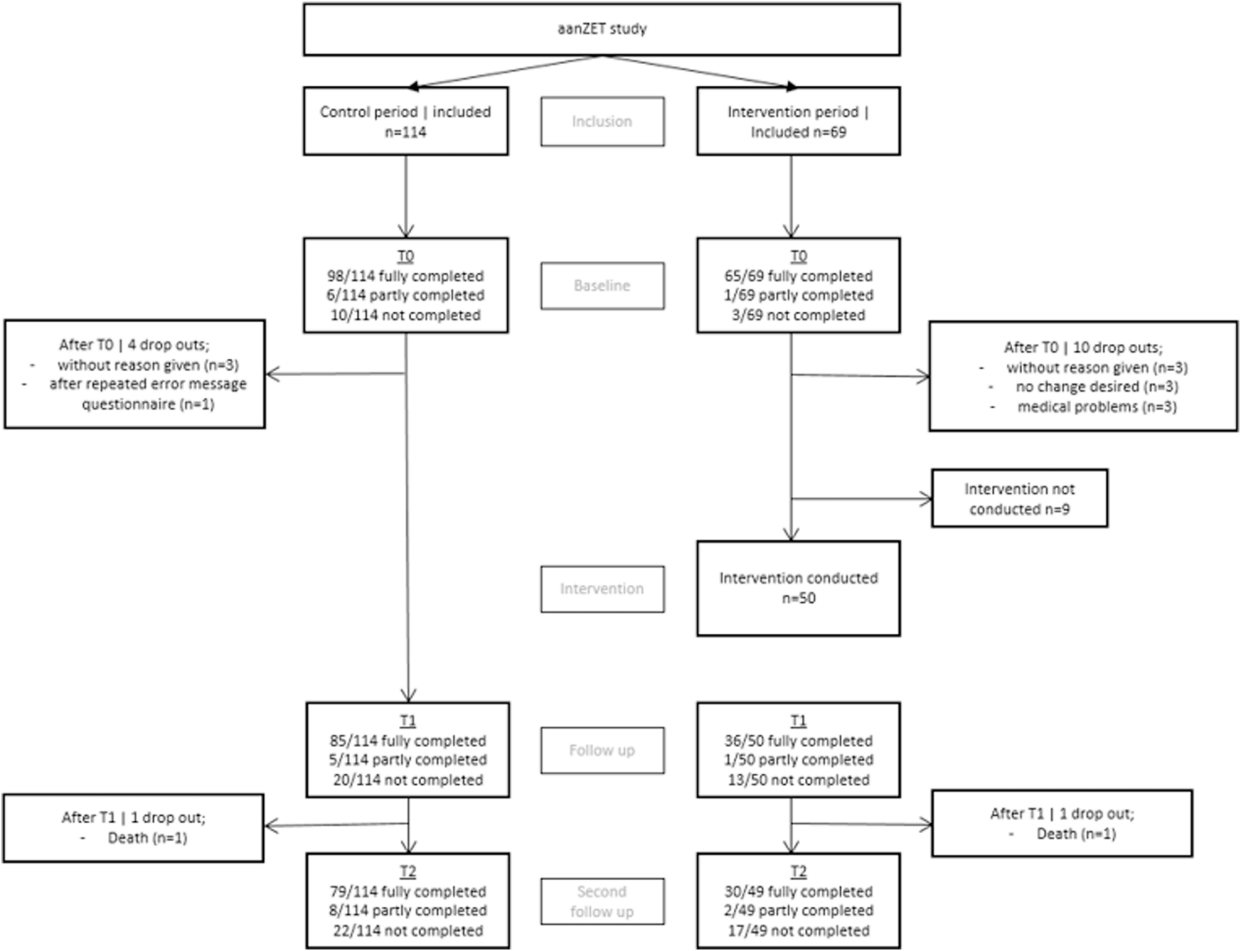
Inclusion and drop-out.

For an overview of the inclusion and drop-out, see Figure 3. All departments included participants during the control group. Due to logistical difficulties two departments were not able to include participants in the experimental group.

### Participants

For an overview of the participants characteristics, see Table 1.

**TABLE 1:** Descriptive characteristics and comparison between control and experimental group.

	Total (n = 172)	Control group (n = 106)	Experimental group (n = 66)	P
Age Mean (SD)	53 (13.7)	53 (13.6)	53 (14.0)	0.906
Sex Male (%)/women (%)	107 (62.9%)/63 (37.1%)	71 (67.6%)/34 (32.4%)	36 (55.4%)/29 (44.6%)	0.111
Educational level Low (%) Middle (%) High (%)	70 (41.2%) 53 (31.2%) 47 (27.6%)	42 (40.4%) 36 (34.6%) 26 (25.0%)	28 (42.4%) 17 (25.8%) 21 (31.8%)	0.420
Organ – multiple response Kidney (%) Heart (%) Liver (%) Lung (%) Pancreas (%)	146 (86.4%) 12 (7.0%) 8 (4.7%) 9 (5.3%) 1 (0.6%)	91 (86.7%) 8 (7.6%) 3 (2.9%) 4 (3.8%) —	55 (83.3%) 4 (6.1%) 5 (7.6%) 5 (7.6%) 1 (1.5%)	
Donor type Living (%) Deceased (%)	98 (57.0%) 74 (43.0%)	57 (53.8%) 49 (46.2%)	41 (62.1%) 25 (37.9%)	0.282
Medication (multiple response) Azathioprine (%) Cyclosporine (%) Everolimus (%) CellCept (%) Prednisolon (%) Rapamycine (%) Tacrolimus (%) Others (%)	3 (1.8%) 6 (3.5%) 9 (5.3%) 140 (81.9%) 134 (78.4%) 1 (0.6%) 160 (93.6%) 2 (1.2%)	2 (1.9%) 4 (3.8%) 5 (4.8%) 85 (81%) 83 (79%) 1 (1.0%) 98 (93.3%) —	1 (1.5%) 2 (3.0%) 4 (6.1%) 55 (83.3%) 51 (77.3%) — 62 (93.9%) 2 (3.0%)	

### Self-Management Skills

At T0, participants in the control group scored significantly higher on the primary outcome heiQ-STA compared to the participants in the experimental group (*p* = 0.02), see Table 2. There was a significant increase in heiQ-STA scores between T0 and T1 in the experimental group (*p* = 0.025) and remained stable over time (T2) (*p* = 0.564). For the control group, no significant difference between T0 and T1 was found (*p* = 0.429). Between T0 and T2 for the control group, a significant decrease was found on the secondary outcome heiQ-HSN (*p* = 0.004). The effect of the intervention could not be significantly demonstrated using the GLM based on the interaction between groups and time (*p* = 0.082), see Table 3. As none of the covariates were significantly related to heiQ-STA, these were not included in the GLM. There were no significant differences between the groups at T1 and T2 on the remaining subscales, see Tables 3, 4.

**TABLE 2:** Univariate analyses of self-management skills, quality of life, self-regulation and evaluation of experience.

Median (IQR)	Control group T0^a^	Control group T1^b^	Control group T2^c^	Exp. group T0^d^	Exp. group T1^e^	Exp. group T2^f^	P-value between a and b	P-value between d and e	P-value between a and c	P-value between d and f	P-value between a and d	P-value between b and e	P-value between c and f
HEIQ – self-management skills	(n = 102)^a^	(n = 94)^b^	(n = 84)^c^	(n = 65)^d^	(n = 39)^e^	(n = 31)^f^							
Skill and technique acquisition	3.0 (2.8–3.5)	3.0 (3.0–3.8)	3.0 (2.8–3.5)	3.0 (2.8–3.3)	3.0 (3.0–3.8)	3.0 (3.0–3.5)	0.429	0.025**	0.507	0.564	0.025**	0.915	0.910
Health-directed activity	3.5 (3.0–4.0)	3.5 (3.0–4.0)	3.5 (3.0–4.0)	3.5 (3.0–4.0)	3.5 (3.3–4.0)	3.8 (3.3–4.0)	0.525	0.362	0.133	0.566	0.923	0.295	0.417
Positive and active engagement in life	3.2 (3.0–3.6)	3.2 (2.8–3.6)	3.2 (3.0–3.4)	3.2 (3.0–3.6)	3.2 (3.0–3.7)	3.4 (3.0–3.6)	0.705	0.427	0.448	0.103	0.531	0.678	0.085
Emotional distress	3.2 (2.8–3.7)	3.2 (2.8–3.7)	3.2 (2.8–3.7)	3.2 (2.8–3.7)	3.3 (3.0–3.7)	3.3 (3.0–3.8)	0.327	0.261	0.982	0.079	0.637	0.251	0.137
Self-monitoring and insight	3.3 (3.1–3.7)	3.3 (3.0–3.7)	3.3 (3.0–3.7)	3.3 (3.0–3.7)	3.3 (3.2–3.7)	3.3 (3.0–3.7)	0.405	0.112	0.568	0.412	0.565	0.810	0.904
Constructive attitudes and approaches	3.4 (3.0–3.8)	3.2 (3.0–3.8)	3.2 (3.0–3.8)	3.2 (3.0–3.8)	3.4 (3.0–4.0)	3.4 (3.0–4.0)	0.256	0.412	0.068	0.251	0.763	0.226	0.186
Social integration and support	3.2 (3.0–3.8)	3.2 (3.0–3.7)	3.2 (3.0–3.6)	3.2 (2.9–3.8)	3.4 (3.0–3.8)	3.0 (3.0–3.8)	0.060	0.459	0.059	0.627	0.444	0.228	0.583
Health service navigation	3.6 (3.0–3.8)	3.5 (3.0–3.9)	3.4 (3.0–4.0)	3.4 (3.0–3.8)	3.4 (3.0–4.0)	3.6 (3.0–3.8)	0.089	0.243	0.004**	0.723	0.320	0.547	0.592

WHOQoL – BREF – quality of life	(n = 98)^a^	(n = 87)^b^	(n = 78)^c^	(n = 65)^d^	(n = 36)^e^	(n = 30)^f^	P-value between a and b	P-value between d and e	P-value between a and c	P-value between d and f	P-value between a and d	P-value between b and e	P-value between c and f
Physical health	15.4 (13.7–17.1)	16.0 (14.3–18.3)	16.0 (13.7–17.7)	14.9 (13.1–16.6)	16.0 (13.7–17.6)	16.0 (14.7–17.9)	0.070	0.239	0.396	0.035*	0.127	0.571	0.494
Psychological	16.0 (14.7–17.3)	16.0 (14.7–17.3)	16.0 (14.0–18.0)	15.3 (14.3–16.7)	15.7 (14.7–16.7)	16.0 (14.7–17.3)	0.130	0.657	0.374	0.761	0.101	0.716	0.730
Social relationships	16.0 (14.3–17.3)	16.0 (13.3–17.3)	16.0 (13.3–17.3)	16.0 (14.7–17.3)	16.0 (14.7–17.3)	14.7 (13.3–17.3)	0.266	0.958	0.015*	0.985	0.546	0.793	0.992
Environment	16.5 (15.0–18.5)	16.5 (15.0–18.5)	16.5 (15.0–18.1)	16.8 (15.5–18.5)	17.5 (14.6–18.5)	16.5 (15.5–18.5)	0.554	0.768	0.420	0.224	0.638	1.000	0.540
Overall perception QoL	4.0 (4.0–5.0)	4.0 (4.0–5.0)	4.0 (4.0–5.0)	4.0 (4.0–4.0)	4.0 (4.0–5.0)	4.0 (4.0–5.0)	0.371	0.394	0.108	0.317	0.521	0.868	0.373
Overall perception of health	4.0 (4.0–4.0)	4.0 (4.0–5.0)	4.0 (4.0–5.0)	4.0 (4.0–4.0)	4.0 (3.0–5.0)	4.0 (4.0–4.0)	0.692	0.648	0.489	0.745	0.249	0.516	0.849

SSIt – Self‐regulation	(n = 102)^a^	(n = 89)^b^	(n = 79)^c^	(n = 65)^d^	(n = 36)^e^	(n = 30)^f^	P-value between a and b	P-value between d and e	P-value between a and c	P-value between d and f	P-value between a and d	P-value between b and e	P-value between c and f
Setbacks	2.3 (1.7–3.0)	2.4 (2.0–3.0)	2.5 (2.0–3.0)	2.4 (2.0–3.1)	2.4 (1.9–2.8)	2.3 (1.9–2.8)	0.017*	0.888	0.042*	0.868	0.401	0.470	0.356
Successes	4.1 (3.8–4.6)	4.1 (3.8–4.6)	4.0 (3.7–4.0)	4.0 (3.9–4.4)	4.2 (4.0–4.6)	4.2 (4.0–4.5)	0.452	0.299	0.041*	0.843	0.503	0.230	0.034*

Evaluation of experience	(n = 83)			(n = 35)			P-value between a and b	P-value between d and e	P-value between a and c	P-value between d and f	P-value between a and d	P-value between b and e	P-value between c and f
Overall experience	—	10 (8.0–10.0)	—	—	10.0 (9.0–10.0)	—	—	—	—	—	—	0.920	—
CAHPS – total score	—	20.0 (18.0–20.0)	—	—	20.0 (18.0–20.0)	—	—	—	—	—	—	0.593	—

*p < 0.05; **p < 0.001. Comparison between a and b, and c and d was conducted using a Wilcoxon signed ranked test. Comparison between a-c and b-d were conducted using a Mann-Whitney U test.

**TABLE 3:** General linear model for self-management skills, quality of life and self-regulation.

	Follow up T1 mean difference (experimental – Control, 95% CI)	P-value*	Follow up T2 mean difference (experimental – Control, 95% CI)	P-value*	P-value for interaction**
HeiQ					
Skills and technique acquisition	−0.013 (−0.189–0.163)	0.886	−0.009 (−0.196–0.178)	0.922	0.082
Positive and active engagement in life	0.036 (−0.158–0.230)	0.714	0.168 (−0.018–0.354)	0.077	0.180
Emotional distress	0.110 (−0.087–0.308)	0.272	−0.161 (−0.061–0.383)	0.153	0.094
Self-monitoring and insight	−0.063 (−0.205–0.079)	0.381	−0.004 (−0.153–0.144)	0.954	0.631
Constructive attitudes and approaches	0.152 (−0.039–0.342)	0.118	0.215 (0.026–0.405)	0.026	0.036*
Social integration and support	0.123 (−0.070–0.316)	0.210	0.094 (−0.102–0.290)	0.346	0.162
WHOQoL-BREF					
Physical health	−0.799 (−1.867–0.269)	0.142	−0.148 (−1.269–0.973)	0.795	0.135
Psychological health	−0.128 (−0.995–0.739)	0.771	−0.073 (−0.969–0.823)	0.872	0.392
Social relationships	0.184 (−0.868–1.236)	0.731	0.319 (-0.751–1.390)	0.556	0.448
Environment	0.109 (−0.776–0.994)	0.808	0.249 (−0.601–1.099)	0.563	0.934
SSIt					
Setbacks	−0.082 (−0.344–0.179)	0.536	−0.121 (−0.151–0.393)	0.379	0.190
Successes	0.106 (−0.093–0.306)	0.293	0.108 (−0.105–0.321)	0.317	0.093

*P-value for the difference in estimated marginal means between the experimental group and the control group.

**P-value for the interaction between time-point and group.

**TABLE 4:** Results of generalized estimating equation models for dichotomized variables of self-management, quality of life and treatment adherence.

	Follow up T1 Odds ratio (95% CI)	P-value*	Follow up T2 Odds ratio (95% CI)	P-value*
HeiQ				
Health-directed activity	0.905 (0.791–1.036)	0.149	1.048 (0.895–1.229)	0.560
Health service navigation	0.969 (0.880–1.068)	0.529	0.940 (0.866–1.020)	0.139
WHOQoL-BREF				
Quality of life assessment	0.908 (0.700–1.178)	0.468	0.755 (0.565–1.010)	0.059
Satisfaction with health	1.014 (0.702–1.463)	0.943	0.848 (0.592–1.214)	0.368
BAASIS				
Adherence vs. non-adherent	1.905 (0.621–5.843)	0.260	1.848 (0.606–0.5.638)	0.280

### Quality of Life

The univariate analysis showed no significant differences in QoL between the groups at the timepoints. A significant improvement after the intervention was found in outcome physical health within the experimental group between T0 and T2 (*p* = 0.035), see Table 2. The GLM and GEE could not demonstrate an effect of the intervention for any QoL scales, see Tables 3, 4.

### Self-Regulation

At T0 and T1, no significant differences between groups were found on the scales Setbacks and Successes. At T2, the experimental group scored significantly higher on the scale Successes compared to the control group (*p* = 0.034). For the control group, an increase was found between T0 and T1 on the scale Setbacks (*p* = 0.017) and between T0 and T2 (*p* = 0.042). For the subscale Successes, the control group scored significantly lower at T2 than at T0 (*p* = 0.041). For the experimental group no significant difference were found between T0, T1 and T2 on self-regulation. The GLM found no significant effect of the intervention between groups and time-points for both scales, see Table 3.

### Medication Adherence

At T0 there was no differences in medication adherence between groups (see Table 5). Similarly at T1 and T2, no significant difference was found on the outcome medication adherence between groups. For the control group, a decrease of medication adherence on the scale Taking was found between T0 and T2 (*p* = 0.038). In addition for the control group, an decrease in medication adherence on the scale Timing was found between T0 and T1 was found (*p* = 0.048). The GEE found no significant effect of the intervention between groups and time-points, see Table 4.

**TABLE 5:** Univariate analyses of medication adherence.

N (%)	Control group T0^a^ (n = 98)	Control group T1^b^ (n = 85)	Control group T2^c^ (n = 77)	Exp. group T0^d^ (n = 65)	Exp. group T1^e^ (n = 36)	Exp. group T2^f^ (n = 28)	P-value between a and b	P-value between d and e	P-value between a and c	P-value between d and f	P-value between a and d	P-value between b and e	P-value between c and f
Medication adherence –overall Adherent (%) Non-adherent (%)	80 (81.6%) 18 (18.4%)	60 (70.6%) 25 (29.4%)	56 (72.7%) 21 (27.3%)	51 (78.5%) 14 (21.5%)	28 (77.8%) 8 (22.2%)	22 (78.6%) 6 (21.4%)	0.134	1.00	0.405	1.00	0.618	0.417	0.545
Medication adherence –taking Adherent (%) Non-adherent (%) One time (%) Two times (%) Three times (%) Four or more times (%) Missing (%)	93 (94.9%) 5 (5.1%) 1 (1%) — — 4 (4.1%) —	76 (83.5%) 9 (10.6%) 9 (10.6%) — — — —	66 (85.7%) 11 (14.3%) 8 (10.4%) 2 (2.6%) — 1 (1.3%) —	62 (95.4%) 3 (4.6%) 3 (4.6%) — — — —	34 (94.4%) 2 (5.6%) 1 (2.8%) 1 (2.8%) — — —	27 (96.4%) 1 (3.6%) 1 (3.6%)	0.118	0.842	0.038*	0.726	0.888	0.379	0.127
Follow up question – drug holiday No (%) One time (%) Two times (%) Three times (%) Four or more times (%) Missing (%)	4 (80%) — — — 1 (20%) —	9 (100%) — — — — —	10 (90.9%) — — — 1 (9.1%) —	3 (100%) — — — — —	2 (100%) — — — — —		—	—	—		—	—	—
Medication adherence – timing Adherent (%) Non-adherent (%) One ime (%) Two – three times (%) About once a week (%) Few times a week (%) Almost every day (%) Missing (%)	80 (85.1%) 14 (14.9%) 8 (8.5%) 5 (5.3%) 1 (1.1%) — — —	64 (75.3%) 21 (24.7%) 12 (14.1%) 6 (7.1%) 2 (2.4%) 1 (1.2%) — —	60 (77.9%) 17 (22.1%) 10 (13.0%) 4 (5.2%) 2 (2.6%) - 1 (1.3%) —	52 (81.3%) 12 (18.8%) 9 (14.1%) 2 (3.1%) 1 (1.6%) — — —	30 (83.3%) 6 (16.7%) 3 (8.4%) 3 (8.4%) — — — —	23 (82.1%) 5 (17.9%) 3 (10.7%) 2 (7.2%) — — — —	0.048*	0.779	0.175	0.910	0.521	0.332	0.638
Reduction of dose Adherent (%) Non-adherent (%)	97 (100%) —	85 (100%) —	77 (100%) —	64 (100%) —	36 (100%) —	28 (100%) —	1.000	1.000	1.000	1.000	1.000	1.000	1.000
Persistence Adherent (%) Non-adherent (%)	97 (100%) —	85 (100%) —	77 (100%) —	64 (100%) —	36 (100%) —	28 (100%)	1.000	1.000	1.000	1.000	1.000	1.000	1.000

*p < 0.05; **p < 0.001. Comparison between a-b, a-c, d-e and d-f were conducted using a GEE. Comparison between a-d and b-e was conducted using a Chi-square test.

### Evaluation of Experience

No significant difference was found between groups on the scale Patient-centeredness, see Table 2. The perceived experience of the nurse-led care measured using the VAS, was considered high with a median score of 10 (IQR 8–10). Most participants (91.2%) of the experimental group indicated that they would recommend the program to peers. Reasons included the fact that it supports setting new goals, achievement of goals, as well as in everyday life after transplantation. Participants also indicated that this program gives insight and tools to help move forward. There were also participants who would recommend the program, but indicated that they did not need it because they did not experience any problems. Some of the participants would not recommend the program.

### Intervention Fidelity

Table 6 shows that most participants received four sessions (84%) of the intervention, and 100% of the participants who received the intervention completed all steps of the intervention. The Self-Management Web was used during most sessions (96%). Most participants received the patient booklet (98%). For all items, intervention fidelity was found to be adequate.

**TABLE 6:** Descriptive statistics of the intervention fidelity.

N (%)	Experimental group T1 (n = 50)
How many intervention sessions did the participant received? One session (%) Two sessions (%) Three sessions (%) Four sessions (%) More than four sessions (%)	— 3 (6%) 5 (10%) 42 (84%) —
Have all the steps been completed? Yes(%)/No(%)/	49 (100%)/0 (0%)
How often was the Self-Management Web used? Never Sometime, but not every session Throughout all sessions	1 (2%) 48 (96%) 1 (2%)
Did the recipient receive the participant booklet during the first session? Yes(%)/No(%)/Don’t know (%)	49(98%)/0(0%)/1 (2%)

## Discussion

In this study, we implemented and tested the ZENN-intervention in a multicenter stepped-wedge RCT among SOTx recipients.

The analyses showed that there were no significant differences in the primary and secondary outcomes at T1, suggesting that there was no effect of the intervention. However, analyses also revealed that the participants in the experimental group were less skilled in self-management when they entered the intervention and that they made significant improvements over time. After the intervention they had reached the same skill level of participants included in the control group. In addition, participants in the experimental group reported worse perceived physical health at baseline which improved over time. Moreover, the experimental group reported greater self-regulation successes at T1 compared to the control group. The differences at baseline are indicative of bias in inclusion, this could be either self-selection bias or bias by those including the recipients. As the control group did not entail participation in an intervention, this may have appealed to a broader audience to consent to participation. It is possible that those who felt the need for SMS were more likely to be approached to participate or agree to participate in the intervention. This may explain the differences at baseline as well as the difference in sample size between the groups. In the future, qualitative research on motivation to participate among recipients and inclusion choices among NPs may help shed light on the cause of this bias. Although, in this study we could not demonstrate a significant effect of the intervention, some findings point to potential of the intervention which require more investigation. It is possible that the intervention is effective in a more selected group of those in need of SMS, whereby a matched control group on self-management skills would offer a better comparison.

In addition, we were unable to include and retain sufficient participants in the experimental group for a sufficiently powered analysis. Three main factors contributed to the low number of inclusions. Firstly, while we implemented inclusion and exclusion criteria, a needs assessment was not part of the recruitment strategy. For example, there were participants who indicated that they thought it was a good program but did not consider it necessary for themselves as they were not experiencing self-management issues. So, some recipients may have been less in need of, and thus less engaged in the intervention. This may also have led to a ceiling effect on the questionnaires. Therefore, when using the intervention, it may be better to include a screening step, for example, using the Self-Management Web. If self-management problems are identified, the intervention could be continued.

Secondly, the COVID-19 pandemic had an impact on the inclusion rate. The study started later due to the pandemic and NPs were given additional duties, for example, temporarily working in the intensive care unit. Consequently, there were staffing shortages and a backlog of work to be caught up on. During the control period, the role of the NPs was to recruit the participants and register them with the investigator. The combination of these administrative tasks with implementing the intervention during the experimental period required a greater time investment which proved challenging in the post-COVID period.

Thirdly, the pandemic also affected the training of the NPs. Initially, the plan was to provide the training in two steps consisting of theory through an e-learning module, and a practical interpersonal skills training in a live group session. Due to the restrictions on visiting other hospitals, this proved impossible. The live training was therefore completed online. It is not clear whether this had adverse effects on the self-efficacy and development of the skills needed to implement the intervention. How NPs experienced this is also unclear. It would therefore be useful to gain insight into this through interviews with those who delivered the intervention.

Research on successful self-management interventions shows that effective support is found in tools such as reminders, medication logs, registration of symptoms, rehabilitation guidance modules, decision support tools and tools for healthcare providers for care assessment [35]. These are practical tools to SMS, while the ZENN-intervention primarily focuses on patient empowerment and skills to set and achieve their own personal goals and take matters in their own hands, with guidance from the NP. This intervention is primarily based on behavior change theories’; it is well established that interventions based on behavior change theories make an important contribution to improving self-management skills in the long term [36–38]. Recipients are stimulated to set goals in the different areas of life. These will not always be health-related such as medication use or monitoring symptoms. Goals can also be, for example, about roles and relationships or solving financial problems. The intervention aims to provide generic skills that can be used for all kinds of goals. The analysis of self-regulation skills shows that at T2 there is a difference between the groups on the success subscale, whereby the intervention group was achieving higher scores on success compared to the control group. This could be an indication that there has been an increase over time in the skills needed to self-manage life. Further research is needed to replicate and confirm the effect.

## Practical Implications and Further Research

In this study, there were differences between groups at baseline which were not expected. Conducting qualitative research among those who implemented the study could help to understand the processes that resulted in these differences and how to avoid this source of bias in future studies. Similarly, qualitative research among participants on their experiences with the intervention and whether this type of intervention matches support needs could be insightful. Suggestions for improvement could be generated as a result.

For the future, it is useful to examine the way in which the intervention or parts of the intervention can be integrated into daily care practice. A possible idea would be to integrate the Self-Management Web within patient dashboards or the Patient Reported Outcome Measures (PROMs) and Patient Reported Experience Measures (PREMs). The Web could act as a starting point for a conversation on self-management and personalized counseling which fits seamlessly with the goals of Value Based Healthcare [39].

Further research could focus on cost-benefit analysis, implementation and evaluation of the intervention and the Self-Management Web among other populations of individuals with a chronic condition. With this intervention, people receive guidance in optimizing skills that are not only useful for recipients after a SOTx and can be of added value in managing life with a chronic disease.

## Conclusion

The analysis demonstrated no effect of the intervention at T1. Secondary analyses demonstrated baseline differences and an increase in self-management skills over time in the experimental group. This suggests that the intervention may be beneficial for a subgroup of transplant recipients with lower self-management skills. Further research will be required to assess which groups of recipients can benefit most from this SMS approach. Participants were generally positive about the program and reported added value.

## Data Availability

The raw data supporting the conclusions of this article will be made available by the authors, without undue reservation.

## Group Members of aanZET Study Group

Erasmus University Medical Center, Rotterdam, Netherlands: Denise Beck, Marleen van Buren, Monique van Dijk, Marleen Goedendorp, Martijn van den Hoogen, Erwin Ista, Marcia Kho, Louise Maasdam, Olivier Manintveld, Emma K. Massey, Marlies Reinders, Joost van Rosmalen, Annelies de Weerd, Regina van Zanten, Robert Zietse; University of Applied Sciences Rotterdam, Rotterdam, Netherlands: Janet Been-Dahmen, AnneLoes van Staa; University Centre for Nursing and Midwifery, Ghent University, Belgium: Ann Van Hecke; Leiden University Medical Center, Leiden, Netherlands: Jeannet Bisschop, Paul van der Boog, Ruth Dam, Tessa van Diemen, Maaike Konijn, Esther Nijgh; Radboudumc, Nijmegen, Netherlands: Marjo van Helden, Luuk Hilbrands; University Medical Center Groningen, Groningen, Netherlands: Coby Annema, Lyda Engelsman, Tally Norder, Christina Oosterhoff, Irma Saro, Geesje Smeenge; University Medical Center Utrecht, Utrecht, Netherlands: Sanne Bosman, Esther de Haan, Anja Kooistra, Arjan van Zuilen.
